# Floridoside Extracted from the Red Alga *Mastocarpus stellatus* Is a Potent Activator of the Classical Complement Pathway

**DOI:** 10.3390/md20080019

**Published:** 2008-07-10

**Authors:** Anthony Courtois, Christelle Simon-Colin, Claire Boisset, Christian Berthou, Eric Deslandes, Jean Guézennec, Anne Bordron

**Affiliations:** 1 Biotechnologies and Marine Molecules laboratory, IFREMER – Brest Center, Technopôle Brest-Iroise, France; E-mails: Anthony.Courtois@ifremer.fr (A.C.); Christelle.Simon.Colin@ifremer.fr (C.S.-C); Claire.Boisset@ifremer.fr (C.B.); Jean.Guezennec@ifremer.fr (J.G.); 2 Cellular Therapy and Immunobiology of Cancer laboratory, EA2216, Brest University Hospital, France; E-mails: christianberthou@wanadoo.fr (C.B.); Anne.bordron@univ-brest.fr (A.B.); 3 Ecophysiology and Biotechnology of Halophytes and marine Algae laboratory (LEBHAM), European Institute of Marine Studies, University of Western Brittany, Technopôle de Brest-Iroise E-mail: eric.deslandes@univ-brest.fr (E.D.)

**Keywords:** Alpha-galactosyl glycerol, marine algae, immunomodulation, complement system

## Abstract

Many biological properties of algae have been found to have useful applications in human health, particularly in the fields of oncology and immunology. Floridoside, extracted from the red alga *Mastocarpus stellatus*, has a structure similar to the xenoantigen Gal alpha 1–3 Gal. This xenoantigen has been described to induce a high immune response in human xenografts and is mediated by natural anti-gal antibodies that activate the classical complement pathway. Based on this property, we analyzed the potential activities of floridoside on the immune system. We demonstrated that floridoside activates a complement cascade via the classical complement pathway, through the recruitment and activation of natural IgM. This algal molecule could represent an important step in the development of a potent new anticomplementary agent for use in therapeutic complement depletion.

## 1. Introduction

Molecules isolated from marine organisms have already been reported to possess antitumoral activities. However, in comparison to sponges, mollusks or ascidians, algae have been at the origin of only few studies focused on oncological or immunological applications. In fact, biological properties of oncological or immunological interest have been demonstrated in 140 species of algae, but molecules from only three species of red algae are currently in preclinical trials. Previous studies have often been limited to preliminary screening without collaboration with pharmaceutical laboratories for developing new drugs. Nevertheless, the discovery, isolation and characterization of new bioactive marine molecules (especially terpenes, polysaccharides, alkaloids, halogenated compounds, phenolic compounds, etc.) from different types of marine algae should generate many productive studies in terms of potentially useful biological properties in the near future.

Floridoside is a neutral heteroside found in many red algae originally isolated from the Rhodophyceae *Rhodymenia palmate* or *Mastocarpus stellatus*. Produced directly from photosynthesis, floridoside constitutes an important soluble carbon reserve readily available according to cellular needs [1,2] and, in aqueous solution, it behaves as an osmoregulator involved in the resistance of the algal cell wall to changes in external salinity. The molecular structure of floridosides was first established by Putman and Hassid [3] in 1954, who characterized it as a 2-*O*-α-D-galactopyranosylglycerol. The terminal Galα (1–3) structure of this molecule is similar to the linear B trisaccharide (Galα (1–3)Galβ (1–4)GlcNAc) named Gal xenoantigen or Galili epitope [4]. This antigen is responsible for the human xenograft rejection due to the presence of natural anti-gal antibodies. These antibodies are mainly immunoglobulins of type M and G (IgM and IgG) with a strong capacity to activate the classical complement pathway, through recruitment and activation of the C1q protein that ultimately leads, to cell destruction by forming the membrane attack complex (MAC).

Because of the structural similarity between floridoside and Galili epitope, we were interested in evaluating the activity of the floridoside on the complement system. First, floridoside was extracted from the red alga *Mastocarpus stellatus*, purified and characterized. Then, its activity on the complement system was tested using hemolytic assays as well as in a solid-phase Enzyme-Linked Immunosorbent Assay (ELISA) system and, finally, compared to Galili epitope activity. The results obtained with floridoside are presented here and the potential biological applications of this molecule are discussed.

## 2. Results and Discussion

### 2.1. Extraction, purification and chemical structure

**Figure 1:**
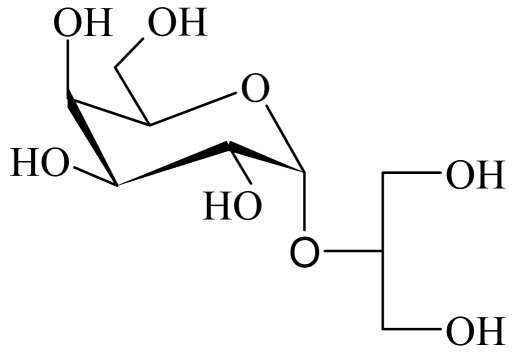
Chemical structure of floridoside.

The floridoside was extracted from the red alga *Mastocarpus stellatus* with a mixture of MeOH-CHCl_3_-H_2_O and was purified from the hydroalcoholic fraction. The chemical structure was determined as C_9_H_18_O_8_ (with a molecular weight of 253 g. mol^−1^) on the basis of NMR and GC/MS analysis. This structure was in complete agreement with those published by Simon-Colin *et al.* [1,2]. Based on these data, floridoside was confirmed as ((2-O-α-D-galactopyranosyl)1→2′ (glycerol)) (Figure 1).

### 2.2. Biological activities of floridoside

The complement system is a major effector of humoral immunity involved in host defense. It is a complex system comprised of at least 30 serum proteins, such as C4 or C2 molecules, which interact in a cascade of activation. Many of the early components are serine proteases, which activate each other sequentially. The activation cascade results in the formation of MAC that perforates the plasma membrane of the target cell [5,6]. In order to determine the role of floridoside on the complement system, hemolytic assays were analyzed after contact with sensitized sheep erythrocytes.

**Figure 2:**
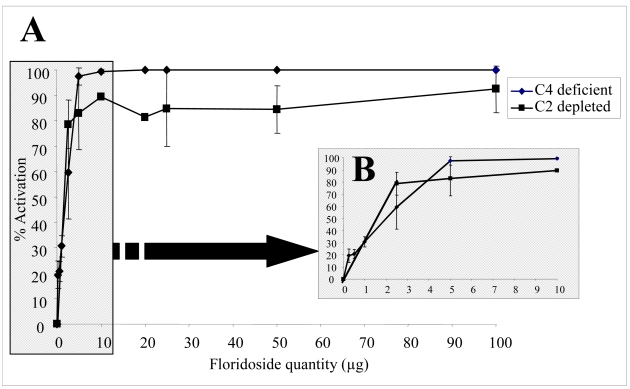
Floridoside activation of the complement system. Activation was measured by the capacity of NHS incubated with various quantities of floridoside to restore serum deficient or depleted in complement proteins C4 and C2, respectively. (A) Activation using a wide range of floridoside concentrations. (B) Close-up of (A) for floridoside quantities ≤10 μg. Each point represents the mean (± SE) determined from three to six trials.

The effect of different floridoside concentrations was determined *in vitro* using normal human serum (NHS) as the complement source. As a first approach, we validated the experimental model by testing different concentrations of aggregated IgG. Aggregated IgG is known to be a powerful activator of the complement system and was used as a positive control for the reaction [7]. The capacity of the NHS serum to restore the hemolytic activity of a serum deficient in one protein of the complement system was measured. First, the CH_50_ representing the NHS dilution that leads to 50% lysis was determined. In our experiments, CH_50_ was reached at a 1:100 dilution. Then, increasing amounts of aggregated IgG (0–100 μg) in CH_50_ NHS conditions were incubated with a C2-deficient serum and the complement activation was determined by measuring the amount of released hemoglobin. The rate of activation by aggregated IgG in veronal buffer (VBS^2+^) increased very rapidly with amounts of IgG and reached a plateau corresponding to 80% (data not shown). These results confirm that aggregated IgG is a very efficient activator. The same experimental conditions were applied to floridoside. Increasing amounts of floridoside (0–100 μg) in CH_50_ NHS conditions were incubated with a C4-deficient or C2-deficient serum and complement activation was determined by measuring the amount of released hemoglobin. As shown in Figure 2A, the rate of activation by floridoside in veronal buffer (VBS^2+^) increased very rapidly with amounts of floridoside and reached a plateau corresponding to 100%. These results indicate that floridoside was very efficient in activating the complement system, with a rate of 50% (AC 50) at 1.5 μg and 2 μg using C2- and C4-deficient serum, respectively (Figure 2B).

**Figure 3:**
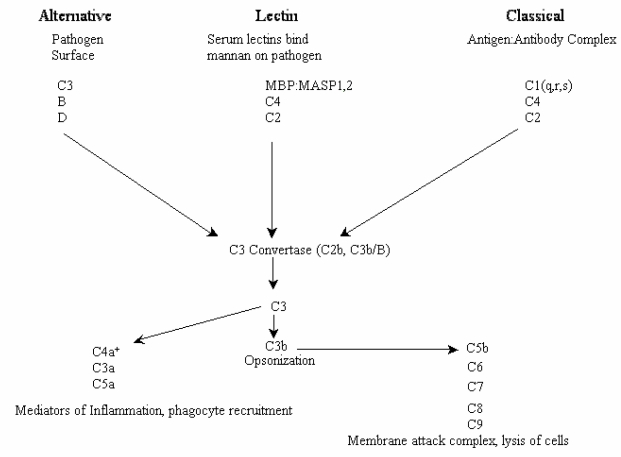
The three main pathways that activate the complement system.

**Figure 4:**
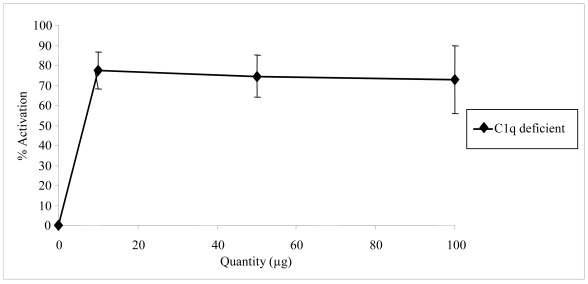
Quantity-response curves of floridoside activation of the complement system. Activation was measured by the capacity of NHS incubated with various quantities of floridoside on restoring serum deficient in complement proteins (C1q). Each point represents the mean (± SE) determined from three to six trials.

Complement activation can be initiated by three pathways, all of them generating homologous variants of the protease C3-convertase. Typically, the classical complement pathway requires antibodies for activation (specific immune response), while the alternative and the mannose-binding lectin (MBL) pathways can be activated either by C3 hydrolysis or by antigens and does not require the presence of antibodies (non-specific immune response) (Figure 3). The C4 and C2 proteins used in our test conditions are specific to both classical and MBL pathways. In order to identify which particular pathway of the complement system was activated by floridoside, additional assays were carried out using a human C1q-deficient serum specific to the classical pathway along with varying amounts of floridoside (10, 50 and 100 μg). The results, presented in the Figure 4, demonstrated that activation occurred using the C1q-deficient serum, indicating that floridoside was specifically involved in the classical pathway.

**Figure 5:**
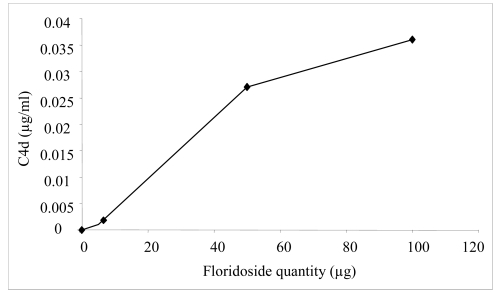
C4d production in NHS incubated with floridoside determined by ELISA assays. Each point represents the mean determined from four trials.

In order to confirm that the classical pathway was specifically activated by floridoside, the production of C4d protein was measured by ELISA assays. Classical pathway activation is triggered upon the binding of the C1q component to IgG- or IgM-containing immune complexes or other activating molecules. The binding of C1q to one of these activators results in the conversion of the C1 complex to an active proteolytic enzyme cleaving C4 to C4a and C4b. As an anaphylatoxin, C4a has a short half-life and is bound by cells possessing the appropriate receptors. C4b mediates opsonization of target cells and can participate in the formation of the classical pathway C3-convertase. C4b is rapidly cleaved by factor I, generating the fragments C4c and C4d. In our experiment, NHS was incubated with various amounts of floridoside (5 μg, 50 μg and 100 μg) and the C4d produced by the complement activation was determined using the Quidel® C4d EIA Kit. Our results showed that floridoside induced the production of C4d in a dosage-dependent way, confirming that it activates the complement system through the classical pathway (Figure 5).

**Figure 6:**
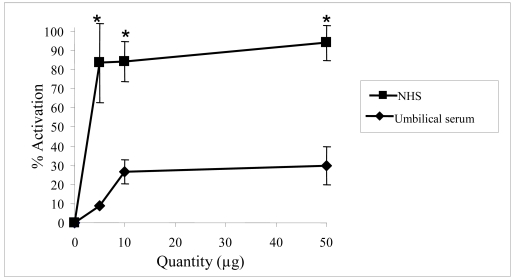
Evaluation of floridoside complement activation using NHS and umbilical serum (US). Experiments were performed using serum deficient in the C4 protein. Each point represents four trials analyzed using the Student’s *t*-test and ANOVA. The asterisk (*) represents significant differences (p<0.05) between NHS and US at a given quantity.

Moreover, the classical pathway can be activated via two main mechanisms: (1) a direct activation of the C1q protein or (2) the natural recruitment of IgM able to activate the system. In order to determine the mechanism of activation, we evaluated the activation of the complement system in a serum obtained from umbilical cord blood (US) in which IgM are lacking [8]. Various quantities of floridoside (5 μg, 10 μg and 50 μg) were incubated with US and the complement system activation was evaluated by hemolytic assays using human serum deficient in the C2 protein. Results were compared to those obtained using NHS (Figure 6). Floridoside activation using US was significantly weaker than the one measured using NHS under the same experimental conditions. This result indicates that activation of the classical complement pathway by floridoside is mediated by natural IgM. The observed residual activity was most likely due to natural IgG directed against floridoside.

**Figure 7:**
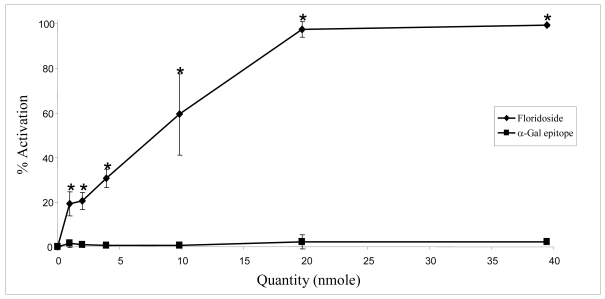
Comparison between floridoside and α-Gal epitope in their capacity to activate the complement system. This experiment was performed using serum deficient in the C4 protein. Each point represents four trials analyzed using Student’s *t*-test and ANOVA. The asterisk (*) represents significant differences (p<0.05) between floridoside and α-Gal epitope at a given concentration.

We can conclude that floridoside constitutes a potent activator of the classical complement pathway and this activation seems to be mediated by the recruitment and the activation of natural IgM directed against this molecule. Floridoside possesses an alpha-Gal terminal structure similar to the Galili epitope. As noted previously, this α-galactosyl core is absent in human cells but is naturally expressed—with millions of epitopes per cell—on glycolipids and glycoproteins in non-primate mammals, prosimians (e.g. lemurs) and New World monkeys (monkeys of South and Central America) [4, 9, 10]. This epitope is responsible for xenograft rejection, via the complement system, by recruiting and activating natural IgM and IgG [11]. Anti-Gal IgM and IgG molecules are the most abundant natural antibodies in humans, constituting about 1% of the total immunoglobulins present in serum [9, 12, 13, 14]. Given the structural similarity of floridoside and this immunogenic epitope, we hypothesized that the activation of the complement system by floridoside could be due to these natural anti-Gal antibodies. We performed experiments to compare the capacity of these two molecules to activate the classical pathway. Hemolytic assays were thus carried out by measuring the restoration of a C4-deficient serum with NHS incubated by various amount of floridoside or Galili epitope (1, 2, 4, 10, 20, 40 μmol). The results, presented in Figure 7, indicate that, unlike floridoside, the Galili epitope is not able to activate the complement system. This confirms that the floridoside molecule is able to activate the complement system through its recognition by natural IgM. Furthermore, the main condition for the initiation of complement activation by α-Gal epitope is its fixation to the cell surface. This hypothesis is confirmed by the use of this epitope alone in therapy and by its capacity to interact with natural immunoglobulins (IgM or IgG) without triggering an immune response [15]. A difference in epitope orientation and accessibility (free or linked) could explain why the Galili epitope in this assay did not induce the expected results [4, 9]. Using gas chromatography coupled with mass spectrometry, we verified that floridoside was not aggregated in solution since aggregates could have conferred the observed biological activity (data not shown). This study clearly demonstrated that activation of the complement system by floridoside is undoubtedly due to natural IgM directed against this molecule. However, we do not yet have direct evidence that these antibodies are natural anti-Galili epitope IgM. Additional studies are required to determine which type of IgM actually interacts with the floridoside and confers the capacity to activate the complement system.

In summary, natural floridoside was purified from the red alga *Mastocarpus stellatus* and identified as a (2-O-α-D-galactopyranosyl)1→2’ (glycerol)). Biological results obtained in this study strongly indicate a close relationship between floridoside and innate immunity. This is the first time that the molecule of floridoside has been described as an activator of the complement system, particularly via the classical pathway. This activation is probably due to natural IgM and our results confirm that immune complexes are formed in solution with floridoside. Substantial efforts are being devoted to develop new drugs for complement inhibition [16]. However, these experimental drugs aim to inhibit complement activation by rapidly consuming enzymatic cascade proteins or by depleting them [17]. Floridoside, as a new natural molecule, may represent a promising new anticomplementary agent. Furthermore, the complement system plays an important role in the immunotherapeutic action of monoclonal antibodies [18], particularly in the treatment of cancer. In combination with monoclonal antibodies, floridoside could be used to enhance the biological activity of new targeted therapies and may thereby be a potentially useful drug for cancer therapy.

## 3. Experimental

### 3.1. Materials

Normal Human Serum (NHS) and Umbilical Serum (US) were obtained from healthy donors (National Blood Transfusion Service, Brest, France). Human complement C1q- and C2-deficient sera were purchased from Calbiochem (La Jolla, CA, USA). Guinea pig complement C4-deficient serum was purchased from Quidel (San Diego, USA). Sheep erythrocytes were commercially available (Eurobio, Paris, France). Rabbit anti-sheep erythrocytes antibodies were purchased from Biomerieux (Paris, France). ELISA C4d quantification kit was purchased from Quidel Corporation (San Diego, USA). Trisaccharide Galα (1–3)Gal-β (1–4)GlcNAc was ordered from Carbohydrate Synthesis (Oxford, UK). IgG was purchased from Roche (Paris, France).

### 3.2. Buffers

The following buffers were used: Phosphate Buffered Saline (PBS) 10 mM, pH 7.4; Veronal Buffer Saline (VBS^2+^): 4 mM veronal (Sigma-Aldrich, Saint-Quentin Fallavier, France), 0.15 mM NaCl (Sigma-Aldrich, Saint-Quentin Fallavier, France), 0.15 mM Ca^2+^ (Sigma-Aldrich, Saint-Quentin Fallavier, France), 0.5 mM Mg^2+^ (Sigma-Aldrich, Saint-Quentin Fallavier, France), pH 7.3. Tris HCl Buffer 10 mM, pH 7.5. Citrate Buffer 0.1 M, pH 3.0.

### 3.3. Extraction and isolation of floridoside

*Mastocarpus stellatus* (Stackhouse) Guiry was harvested from the mid-sublittoral zone at Plouzané, in the Bay of Brest, France. Fresh material was washed with distilled water and frozen in liquid nitrogen. [Frozen] plant material (200 g) was ground in liquid nitrogen and then extracted with a mixture of 12:5:3 MeOH-CHCl_3_-H_2_O (400 mL) for 1 h at room temperature. The hydroalcoholic phase was concentrated in a rotary evaporator, and then purified by passing it through successive columns of AG50 (200 mL, 20–50 mesh, X8, H^+^, Biorad) and AG1 (200 mL, 20–50 mesh, X8, OH^−^, Biorad). The neutral effluent was evaporated to dryness and redissolved in hot EtOH. White crystals of floridoside (500 mg) were obtained after partial evaporation of the alcoholic fraction.

### 3.4. Capacity of normal human serum to lyse 50% of sensitized erythrocytes through the classical pathway (CH_50_ assay)

Antibody-sensitized sheep erythrocytes (AE) were prepared by incubating sheep erythrocytes with rabbit anti-sheep erythrocytes antibodies (Biomerieux, Paris, France) as described by Kazatchkine [19].

The CH_50_ represents the NHS concentration that leads to 50% AE lysis. To determine CH_50_, different concentrations of NHS (800 μL), in VBS^2+^, were incubated with 200 μL of AE at 10^8^ cells. mL^−1^ for 45 min at 37°C. The controls corresponding to 0% (L_0_) and 100% (L_100_) lysis were obtained by incubation in the same conditions with 800 μL of VBS^2+^ and 200 μL of AE. After dilution in cold 0.15 M NaCl (2 mL) solution (except the one corresponding to the L_100_, where 2 mL of double distilled water (DDW) were added) and centrifugation, the residual CH_50_ units of the supernatants, corresponding to lysed EA hemoglobin, were determined by measuring the optical density (OD) at 414 nm (Uvikon, Serlabo, Bonneuil/Marne, France) [20].

### 3.5. Hemolytic assay for evaluation of the classical complement pathway activation

For the evaluation of the capacity of the floridoside to activate the complement system, various amounts of floridoside (0 to 100 μg) were pre-incubated with 15 μL of NHS (1/20 in VBS^2+^) for 45 min at 37°C. Then, a mixture of 100 μL of deficient serum in C1q, C2 or C4 proteins (whose dilution factor was previously determined to obtain 90% cell lysis in these experimental conditions) and 100 μL of AE at 10^8^ cells. mL^−1^ were added and incubated for another 45 min at 37°C. The controls L_0_ and L_100_ were obtained as previously described. After dilution with cold 0.15 M NaCl (2 mL) and centrifugation, the amount of the supernatant hemoglobin released was assessed by measuring OD at 414 nm. A positive control for the reaction was obtained by incubation of aggregated IgG in the same experimental conditions [7]. For the comparison of the floridoside and the trisaccharide Galα (1–3)Galβ (1–4)GlcNAc activity, various concentrations of each molecule (0–40 μmol) were treated under the same experimental conditions.

### 3.6. ELISA for the detection of complement activation products: Quantification of the C4d protein

In order to evaluate complement activation by ELISA assays, the C4d protein production was measured. Briefly, various quantities of floridoside or aggregated IgG (0–100 μg) were incubated with NHS (1/25 in VBS^2+^) for 45 min at 37°C. The amount of C4d was quantified using the commercial kit from Quidel (San Diego, USA), which was used according to the manufacturer’s instructions.

### 3.7. Statistical analysis

All results are expressed as means ± standard error. Statistical differences between experimental groups were determined by ANOVA and individual means were compared with Student’s *t*-test using InStat (GraphPad Software, San Diego, CA, USA). Values of *p* < 0.05 were considered statistically significant.
